# Lifestyle counselling as secondary prevention in patients with minor stroke or transient ischemic attack: a randomized controlled pilot study

**DOI:** 10.1186/s40814-024-01478-4

**Published:** 2024-03-22

**Authors:** Jacob Liljehult, Stig Molsted, Tom Møller, Dorthe Overgaard, Thomas Christensen

**Affiliations:** 1 Department of Neurology, Nordsjællands Hospital, Dyrehavevej 29, Hillerød, 3400 Denmark; 2 Department 9701, The University Hospitals Centre for Health Research (UCSF), Copenhagen University Hospital, Rigshospitalet, Blegdamsvej 9, Copenhagen, 2100 Denmark; 3 Faculty of Health and Technology, Department of Nursing and Nutrition, Copenhagen University College, Tagensvej 86, Copenhagen N, 2200 Denmark; 4 Department of Clinical Research, Nordsjællands Hospital, Dyrehavevej 29, Hillerød, 3400 Denmark; 5 Department of Clinical Medicine, University of Copenhagen, Blegdamsvej 3, Copenhagen N, 2200 Denmark; 6 Department of Public Health, University of Copenhagen, Øster Farimagsgade 5, Copenhagen K, 1353 Denmark

**Keywords:** Adherence, Early rehabilitation, Exercise, Health counselling, Physical activity, Smoking, Stroke, Transient ischemic attack

## Abstract

**Background:**

Patients with minor stroke or transient ischemic attacks have an increased risk of future strokes. These patients are often discharged home with limited specialized follow-up, although close to half of them experience cognitive deficits. Simple encouragements to avoid smoking, be physically active, and to take preventive medication are often insufficient to ensure adherence and more comprehensive interventions are needed to support the patients in adapting healthy behaviour. The aim of this study was to test the feasibility and potential effect of an early initiated, patient-centred intervention to patients with minor stroke or transient ischemic attacks targeting smoking, physical activity, and medication adherence, in a randomized, controlled pilot trial.

**Methods:**

Hospitalized patients were randomized to usual care or an intervention consisting of health behavioural counselling based on the 5A’s model, telephone follow-up (4 and 8 weeks), and monitoring of physical activity. Follow-up time was 12 weeks. Feasibility was on the following domains: eligibility, acceptance, demand and practicality, adherence, attrition, and implementation and integration.

**Results:**

Forty patients of 84 potentially eligible were randomized to the two treatment arms (20 intervention/20 usual care). Thirty-two completed the 12-week follow-up, while 8 were either excluded or lost to follow-up. With few changes, the intervention was feasible and possible to deliver according to the protocol.

**Conclusion:**

It was possible to identify relevant patients who could potentially benefit from a behavioural intervention, recruit and randomize them early after admission and retain most participants in the study until follow-up and derive statistical estimates to guide the design of large-scale randomized controlled trials.

**Trial registration:**

ClinicalTrials.gov Identifier: NCT03648957. Registered 28 August 2018.

**Supplementary Information:**

The online version contains supplementary material available at 10.1186/s40814-024-01478-4.

## Key messages regarding feasibility

- We examined the feasibility of patient-centred counselling focusing on smoking cessation, physical activity, and adherence to preventive medication in a fast-track care pathway for patients with minor stroke or transient ischemic attacks who were discharged home.
- The structured theory-based counselling approach proved to be feasible in the early diagnostic stages and within a hospital-based environment; however, inclusion of patients and provision of the intervention within the short time frame of the fast-track care pathway were found to be challenging, and some elements of the protocol had to be omitted. The use of activity trackers to encourage physical activity required more training and support than anticipated.
- The counselling programme will be feasible to administer in a sufficiently powered trial; however, the screening and inclusion process will have to be optimized.

## Background

The rate of recurrent strokes is high among stroke survivors with approximately 12% of all stroke victims experiencing a new stroke within the first year and one in four patients admitted with a stroke having previously had a stroke or transient ischemic attack (TIA) [1]. Potentially modifiable risk factors, including hypertension, smoking, physical inactivity, abdominal obesity, and unhealthy diet, all contribute to the risk of stroke [2, 3]. Preventive medication, such as antithrombotics, antihypertensives, and statins, is also important in the prevention of recurrent stroke [4], although evidence suggests that adherence to preventive medication among stroke survivors decrease with time [5–8].

Behavioural interventions have previously demonstrated a beneficial effect on hypertension and blood lipids and can potentially be used as part of the secondary prevention, but we lack knowledge as to which approaches are preferable or most efficient in preventing new strokes, beyond the beneficial effect of physical exercise on hypertension [9]. The 5A’s model [10, 11] and Motivational Interviewing [12] are commonly used models for health counselling which have both been validated for use in comparable patient populations. The 5A’s model is a structured approach for facilitating the patient’s personal reflection and goal setting. Motivational interviewing use a person-centred approach which emphasizes acceptance and focus on the needs of the patient and the patient’s life situation as key elements of health counselling.

Most patients with stroke experience minor or transient neurological deficits and are discharged home without significant disabilities. Yet, close to half of patients with minor stroke or TIA report having subtle cognitive and communicative problems, predominantly problems with memory, fatigue, reading, and participating in conversations 3 months after discharge [13]. These impairments along with other factors might influence the stroke survivors ability to manage their own health [14]. We therefore need more research on how behavioural interventions can support the patients’ needs related to improving self-management strategies in secondary and tertiary prevention following stroke.

The aims of this study were to evaluate the feasibility of a patient-centred counselling intervention focused on smoking cessation, physical activity, and adherence to preventive medication in transitional care of patients with minor stroke or TIA and the feasibility of potential outcome measures.

## Methods

We conducted a parallel group randomized controlled pilot and feasibility trial to test the feasibility of implementing an individual face-to-face health behavioural counselling with post-discharge follow-up sessions against usual care in a fast-track stroke care setting. A protocol article outlining the study design and procedures has previously been published [15].

### Setting and participants

The target population was patients hospitalized with recent minor stroke or TIA who were discharged home. We included patients admitted to the Department of Neurology at Nordsjællands Hospital, Denmark, from October 2018 to January 2020, with minor stroke (*ICD-10* I61, I63, I64; Scandinavian Stroke Scale 45–58) or TIA (*ICD-10* G45.9) within the previous 7 days. In Denmark, all patients suspected of acute stroke or TIA undergo admission through a standardized fast-track patient pathway. This pathway includes rapid diagnostic procedures, treatment, and evaluation of risk factors for recurrent strokes. Typically, patients are hospitalized for 3 days. Patients were eligible if they were ≥ 18 years old, could consent to participation, and were discharged home. Exclusion criteria were severe communication barriers, inability to use a telephone, severe disability prior to the stroke (WHO performance status > 2; incapable of self-care and mobilized less than 50% of the day), active abuse of alcohol or narcotics, severe psychiatric illness (affective disease, dementia, schizophrenia), or inability to participate due to severe medical frailty. All new patients with suspected stroke or TIA were screened for eligibility by the primary researcher (J. L.). Potential participants were invited through verbal and written information before discharge. All participants gave written informed consent before participation.

A sample size of 40 randomized participants was chosen weighing the precision of the feasibility measures against the available resources. With this sample size, a theoretical attrition rate of 10% could be estimated with a precision of 3–24% (95% confidence) and a theoretical adherence rate of 5% with a precision of 1–17% (95% confidence) using the Exact method [16].

### Procedures

#### Baseline assessment

Demographic and health behavioural information was collected using standardized questions from the Danish National Health Survey [17], in addition with assessment of stroke severity [18, 19], prior health problems [20], vital signs [21], spirometry (FEV1/FVC) [22], and biochemistry (HbA1c and blood lipids) [4].

#### Randomization and group allocation

Participants were allocated to either intervention or usual care after baseline testing using a simple non-stratified 1:1 randomization. A computer-generated (using the *rbinom*-function in R [23]) randomization sequence was implemented into the Research Electronic Data Capture (RedCap) software [24], which secures concealment of future allocations and prohibits post hoc changes to the allocation. The participants remained in the allocation group for the entire study period.

#### Intervention

**Fig. 1:**
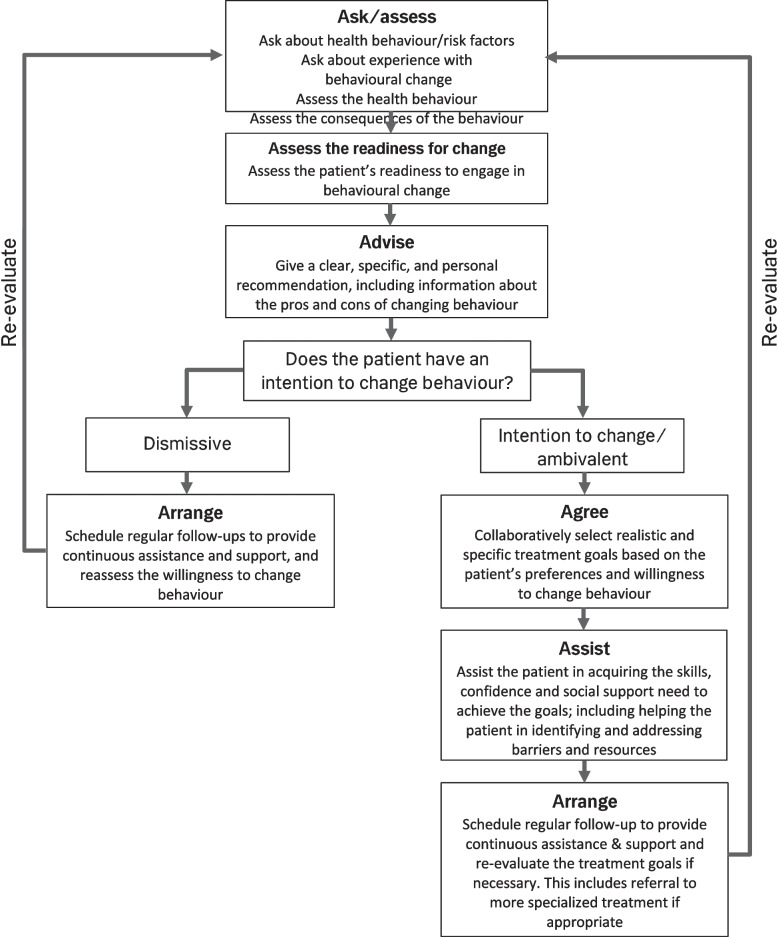
The 5A’s approach modified from Vallis et al. (2013) [10] and Sherson et al. (2014) [11], as proposed in Liljehult et al. (2020) [15]

In brief, participants randomized to the intervention arm received nurse-led targeted health behaviour counselling focusing on smoking cessation, physical activity, and adherence to preventive medication, in addition to usual care. When possible, we tested the participants aerobic capacity prior to the initial counselling using the Astrand-Rhyming cycle test [25]. The initial counselling session was provided face to face by the primary investigator before discharge from the hospital. The counselling employed a patient-centred approach [12] and was structured around the 5A’s model [10, 11] (Fig. 1) with the intention of engaging the participant in healthy behaviour and adhering to preventive medication to reduce their risk of recurrent cerebrovascular events. Participants in the intervention arm were issued a VivoFit activity tracker to count daily steps and aerobic walking time.

Post-discharge counselling was provided by J. L. via telephone 4 and 8 weeks after discharge and aimed at maintaining motivation and adjusting goals and strategies if necessary. A standardized question guide was used to ensure the consistency of the telephone counselling with questions on overall well-being, persistence of symptoms or functional deficits, adherence to medication, side effects, physical activity, smoking, and use of health care services. The intervention is described in details in the published protocol [15].

#### Usual care

Participants in the control arm received standard care, which included a review of prescribed medication, and verbal and written encouragement to cease use of tobacco, diminish the intake of alcohol, be physically active to the extent possible, and eat a healthy diet (reduce the consumption of red meat and salt and increase the consumption of fish, fruit, and vegetables) [26].

### Outcome measures

The primary outcomes were measures of feasibility: (1) *eligibility* including the eligibility rate (proportion of eligible patients compared to the total number of stroke patients), (2) *acceptance* including the study participation rate (proportion of patients accepting participation in the study), (3) *demand and practicality* (proportion of study elements that could be delivered according to the protocol), (4) *adherence* including the degree of adherence to the programme (proportion of attendance in follow-up sessions), (5) *attrition* (drop-out and drawn-out), and (6) *implementation and integration* evaluation the ability of delivering the intervention per protocol.

Secondly, we tested the feasibility of potential clinical outcome measures for use in future studies including the following: arterial blood pressure measured in accordance with US guidelines [27]; self-reported smoking, physical activity level (International Physical Activity Questionnaire-Short Form [28, 29]); adherence to medication (number of missed/consumed doses in the past 7 days); anthropometrics (body weight, waist/hip ratio); fatigue (Fatigue Assessment Scale [30]); long-term readmission with stroke, TIA, or ischemic heart disease; and fatality within 1 year.

#### Follow-up

Participants from both allocation groups were reassessed by the primary investigator (J. L.) in a hospital-based outpatient clinic 12 weeks after discharge. To evaluate the feasibility of potential outcome measures, we measured the following: arterial blood pressure, body weight, and waist/hip ratio, fatigue, smoking, physical activity, and adherence to prescribed medication at 12 weeks. Information on 1-year mortality and stroke recurrence was obtained from electronic hospital records.

### Analysis

We used the model for designing feasibility studies in preventive medicine proposed by Bowen et al. [31] to evaluate the feasibility. The *eligibility rate* was calculated as the proportion of all screened patients who met the eligibility criteria and were available for invitation. The *acceptance rate* was calculated as the proportion of invited patients who accepted participation, in contrast to patients who declined participation or cases where the invitation to participate was retracted before inclusion. *Practicality* was calculated as follows: (1) the proportion of included participants who completed the baseline measurements (baseline interview, SSS, spirometry) or (2) the proportion of participants allocated to the intervention arm who completed the initial elements of the intervention (VivoFit, face-to-face counselling, Astrand-Rhyming cycling test). The *adherence* and *attrition rates* were calculated as follows: (1) the proportion of participants in the intervention arm who participated in the follow-up telephone counselling (4 and 8 weeks after discharge) and used the VivoFit, in contrast to participants who were either drawn out or unavailable/non-adherent, and (2) the proportion of all participants who completed the 12-week follow-up and were included in the 1-year follow-up, in contrast to participants who were either drawn out or lost to follow-up. The *implementation rate* was calculated as the proportion of participants in the intervention arm who completed all elements of the original protocol or a modified version (omitting the Astrand-Rhyming cycling test) in contrast to participants who were non-adherent with one or more elements and the proportion of the participants in the control arm who completed the control protocol in contrast to participants who did not complete the 12 week follow-up. Specific progression criteria were not specified a priori — but the progression of the study was monitored through regular meetings with the programme steering committee at which data on recruitment, protocol adherence, and follow-up were reported and discussed in plenum. Confidence intervals of all proportions were calculated using the Exact method (Clopper-Pearson).

Data was collected in real time using RedCap electronic case report forms. Statistical analyses were carried out in R 3.3.1/R Studio 0.99.

### Ethical considerations

The study was conducted in accordance with the Helsinki Declaration [32] including respect for the participants’ autonomy and right to informed consent. The participants were informed that participation was voluntary, and that further participation could be declined at any time without explanation.

The study protocol was approved by the Scientific Committee of the Capital Region (H-17040484) and the Danish Data Protection Agency (j.nr. VD-2018–306, I-6552). The study protocol was registered at ClinicalTrials.gov (NCT03648957).

## Results

Forty patients accepted participation and were randomly assigned to the two treatments arms (20 intervention and 20 usual care, Table 1 presents the study population characteristics). The median follow-up time was 85 days (range 82–104 days).

**Table 1:** Baseline study population characteristics

	**Intervention ***n* = 20	**Usual care ***n* = 20
Age (years)	66.1 ± 9.3	68.0 ± 6.3
Sex (n/% female)	5 (25%)	6 (30%)
Diagnosis (IS/TIA)^a^	12/6 (60%/30%)	11/8 (55%/40%)
**Scandinavian Stroke Scale** (SSS)		
58	15 (75%)	17 (85%)
57	3 (15%)	0
56	1 (5%)	2 (10%)
55	1 (5%)	0
54	0	1 (5%)
**Living arrangements**		
Living alone	3 (15%)	5 (25%)
Living with a partner	17 (85%)	15 (75%)
**Educational attainment**		
Secondary school	7 (35%)	3 (15%)
Vocational education or training	2 (15%)	8 (40%)
Bachelor’s degree or equivalent	6 (15%)	4 (20%)
Master’s degree	3 (5%)	5 (25%)
**Pre stroke performance status**		
0 (asymptomatic)	18 (90%)	17 (85%)
1 (some symptoms, no disabilities)	2 (10%)	3 (15%)
**Self-rated health**		
Less good	3 (15%)	0
Good	12 (60%)	7 (35%)
Very good	4 (20%)	13 (65%)
**Comorbidities**		
Charlson Comorbidity Index		
*0*	10 (50%)	10 (50%)
*1*	5 (25%)	9 (45%)
*2*	3 (15%)	0
> *2*	2 (10%)	1 (5%)
Known diabetes	4 (20%)	0
Previous stroke	4 (20%)	4 (20%)
Previous myocardial infarction	2 (10%)	1 (5%)
Heart arrythmia (known or diagnosed)	2 (10%)	3 (15%)
**Risk factors**		
Smoking		
*Current*	3 (15%)	0
*Former smoker*	11 (55%)	9 (45%)
*Never smoked*	5 (25%)	11 (55%)
Package years	10 (*IQR* 3–15)	10 (*IQR* 3.9–10.5)
Alcohol intake (units/week)	6 (*IQR* 1.75–20.25)	5 (*IQR* 2.5–8.5)
**Body composition**		
Body weight (kg)	88.5 ± 15.5	82.6 ± 13.6
Body mass index (kg/m^2^)	26.9 (*IQR* 25.5–30.9)	25.5 (*IQR* 23.5–28.1)
Waist/hip ratio	1.00 ± 0.09	0.98 ± 0.12
**Biochemistry**		
HbA1c, mmol/L	7.05 ± 2.15	6.51 ± 0.66
Total cholesterol, mmol/L	4.96 ± 1.16	5.16 ± 1.05
LDL, mmol/L	2.76 ± 0.97	3.09 ± 0.87
HDL, mmol/L	1.38 ± 0.50	1.34 ± 0.30
VLDL, mmol/L	0.71 ± 0.25	0.75 ± 0.35
Triglycerides, mmol/L	1.62 ± 0.63	1.90 ± 1.11

Measures are presented as *mean* ± *standard deviation*, *number (%)*, or *median (interquartile range; IQR)*

*IS* Ischemic stroke, *TIA* Transient ischemic attack, *IQR* Intra-quartile range, *HbA1c* Haemoglobin A1c, *LDL* Low-density lipoprotein, *HDL* High-density lipoprotein, *VLDL* Very low-density lipoprotein

^a^Please note that in three cases, the diagnosis was changed to a final non-stroke diagnosis after the patient was included in the study, which is why the numbers do not add up to 100%

### Feasibility

#### Eligibility and acceptance

**Fig. 2:**
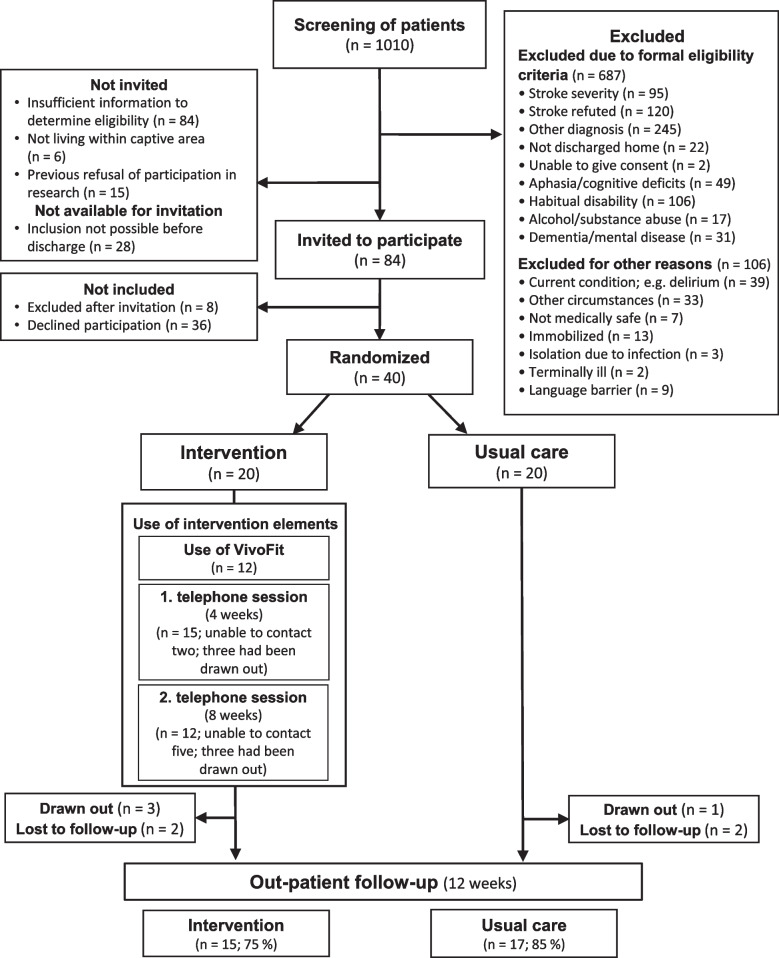
Patient flow from screening to final follow-up

A total of 1010 patients hospitalized in the acute stroke ward were screened for eligibility, of whom 84 were invited to participate in the study. Common reasons for non-eligibility can be seen in Fig. 2. Forty patients accepted participation, while 8 were excluded before inclusion and 36 declined participation. Exclusion after invitation to participate was due to the patient being discharged before consent was obtained (*n* = 4), the stroke diagnosis being refuted (*n* = 2), or changes in the patients’ condition (new stroke or delirium). Reported reasons for declining participation included the following: not finding the intervention to be relevant (perceiving their lifestyle to be healthy already (*n* = 5); not finding their stroke to be associated with their behaviour [*n* = 4]), already having regular contact with health professionals (*n* = 2), time constrains due to work/family obligations (*n* = 5), not having sufficient energy to participate in anything beyond the standard treatment (*n* = 15), while five patients did not specify a reason. Twenty-eight patients were unavailable for invitation due to practical clinical (see Fig. 2).

#### Demand and practicality

**Fig. 3:**
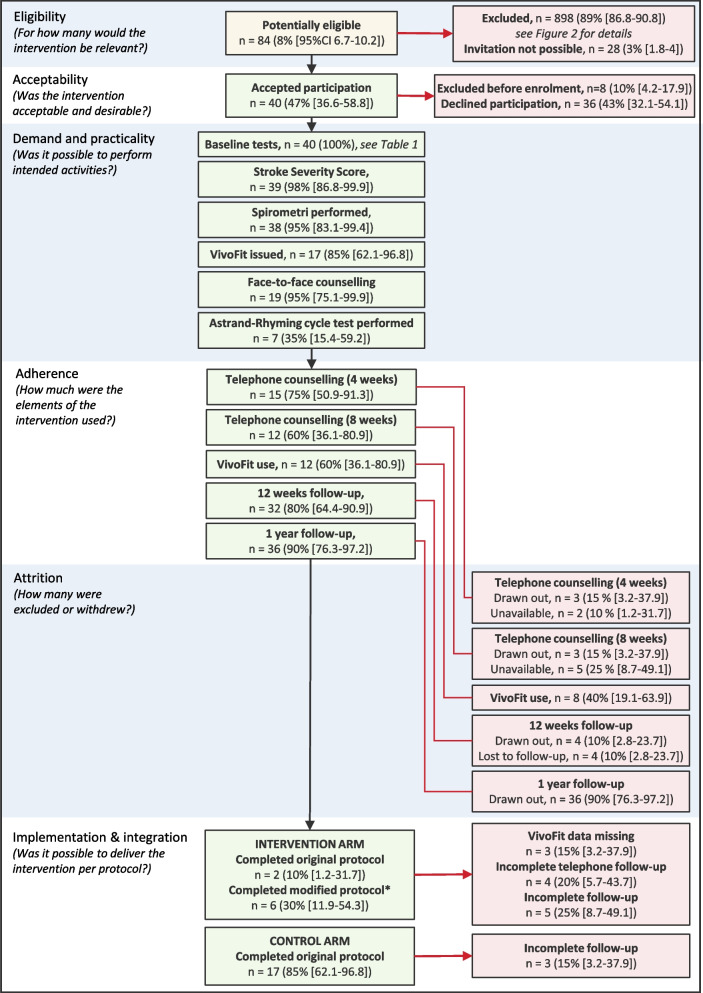
Evaluation of the feasibility

In general, the baseline procedures (SSS, spirometry, and face-to-face counselling) were carried out in high accordance with the protocol (> 95%) (Fig. 3). Setting up the activity tracking devices (VivoFit 4.0) proved to be more time-consuming than expected. All participants needed assistance with installing the smartphone application, setting up a user profile, connecting the device, or support with transferring data to the application after discharged.

In just seven intervention cases (35%) were we able to perform the Astrand-Rhyming cycle test at baseline due to time constrains, and most participants did not find it relevant in relation to the counselling. In addition, two participants declined performing the test due to knee pain.

#### Adherence

In the intervention group, we were able to contact 15 participants after 4 weeks and 12 after 8 weeks for telephone follow-up (Figs. 2 and 3). We received activity tracker data from 12 participants of the intervention group (60%). Two participants wanted to use their own devices (no data returned), one was drawn out before the activity tracker was issued, and five never transferred any data. Among the participants who transferred data, the activity tracker was worn in 90.0% of the days (959 applicable measurements per 1066 follow-up days, 95% *CI* 88.0–91.7) of the intervention period with 4 participants wearing it all days (range 62–100%; see Additional files 2 and 3). Evaluation of the activity tracker data showed a substantial variation in steps per day and aerobic steps per day both within and between subjects. In four participants, the data showed a clear central tendency, while the rest had several clusters throughout their range of activity which might resemble multiple concurrent patterns in the individual activity.

#### Attrition

Thirty-two patients completed the 12-week follow-up period, resulting in an attrition rate at 20% (5 intervention/3 usual care group). Four participants were drawn out after randomization: One was discharged before the intervention had been provided, three had a change of diagnosis after magnetic resonance imaging, and 4 were lost to follow-up. Two follow-up sessions had to be conducted via telephone due to COVID-19 pandemic restrictions at the hospital, and in these, we used blood pressure measurements performed by the patient or the general practitioner.

#### Implementation and integration

We intended to recruit all participants before discharge and integrate the intervention pragmatically within the existing standard treatment. In general, the protocol was manageable within the clinical setting, although we did identify some challenges. In 84 cases, the initial medical records lacked sufficient information to evaluate the eligibility of the patients, and acquiring the information proved to be time-consuming. All participants were discharged home without the intervention causing delay of care.

### Content and feasibility of the counselling

Nineteen intervention participants (95%) received face-to-face counselling, while 15 (75%) and 12 (60%) received subsequent telephone counselling after 4 and 8 weeks, respectively. Medication adherence was discussed with all participants. Two participants had not taken any medication before, and nine had changes to their medication which urged them to change previous habits, while four had no changes to their medication. Four reported being ambivalent towards taking medication. Eighteen participants (90%) received counselling in increasing the level of physical activity. Walking was the preferred type of activity (*n* = 12), while other types included biking, yoga, fitness, and running. Three participants (15%) were current smokers and were recommended to stop smoking: one was already following a cessation course, while the last two intended to stop smoking. Three participants asked for dietary recommendations, which was supplied at their discretion, although this was not part of the protocol.

The 5A’s model organizes the counselling session into a sequence of distinct phases. The model provided rigorous structure to the sessions and guided the direction of the conversation. Each of the distinct phases have a specific function in the session and gives varying emphasis to the contribution of the patient and the health professional. This helped the counselling become a two-way conversation with balanced contributions from the patient and the health professional. Most participants found it difficult to set specific and measurable goals.

### Feasibility of the outcome measures

Measurement of the potential outcome measures was possible according to the study protocol for all participants who came to the outpatient clinic after 12 weeks (estimates per allocation group at baseline and follow-up are available in Additional file 1).

Arterial blood pressure and body composition measures (weight, body mass index, and waist-to-hip ratio) were easily obtained without discomfort or nuisance for the participants. We did encounter some challenges with obtaining the patient-reported outcome measures — mainly that the participants found it hard to recall their level of physical activity and if they had missed any doses of medicine within the last week. Some participants reported using less time on physical activity, although they found it hard to assess if the intensity of activities had changed. The fatigue score was applicable but did require some facilitation from the researcher. Overall, the participants reported feeling more fatigued with the most noticeable difference in the item “Mentally, I feel exhausted”. Potential readmissions and fatalities could be tracked through the electronic patient registry, although only two readmissions were observed.

## Discussion

This study found that it was possible to identify relevant patients early after hospital admission, to randomize participants, and to retain them in the study until follow-up. Few, yet appreciable, changes had to be made to the study protocol, but the overall setup and the structured theory-based counselling approach proved to be feasible in the early diagnostic stages and within a hospital-based environment.

The overall goal of the intervention was to support patients in caring for their own health following a minor stroke or TIA. This included providing knowledge about the disease and behavioural risk factors, the ability to set realistic goals and identify strategies for obtaining these goals, the ability to find relevant help and support, and support of confidence through encouragement and recognition.

The early recruitment of participants allowed for a large part of the target population to be identified. Implementing this in a fast-track patient pathway, however, resulted in potentially relevant patients being unavailable for recruitment and participants being excluded due to the stroke diagnosis being revoked. All patients admitted to the ward were assessed in order to capture as many eligible patients as possible and estimate the potential demand for the intervention. As a result, many non-eligible patients were screened and excluded. Even though stroke mimics were excluded from this study — some did present significant vascular risk factors, and in clinical practice, health behavioural counselling might in fact be beneficial.

The rationale behind including the cycle test and spirometry in addition to the counselling was to facilitate reflection on potential consequences of health behaviour and ensure the participants in the safety of being physically active after a stroke. However, the feedback from the participants indicated that these elements did not have the intended effect, with some participants even refusing to undergo the cycle test due to musculoskeletal conditions.

Previous research has proposed that using a well-structured approach for health behavioural counselling might be helpful to health professionals [33]. For the counselling in this intervention, we used the 5A’s model [15] to provide structure, in combination with techniques from Miller and Rollnick [12], which facilitated a more balanced dialogue between the patient and the health professional. The 5A’s model emphasizes that health recommendations should be specific and personal. This supports the finding of Reed et al. [34] that patients with stroke found it easier to acquire health information if it was connected to their current life situation. Health counselling might therefore be more efficient if it specifically targets problems relevant for the individual patient and is provided in a way that takes the patient’s life situation into account.

Providing effective health counselling requires the health professional to have relevant knowledge to give specific recommendations and make situated clinical decisions, which requires knowledge about the recipient and the context in which the behaviour is performed [35]. In this study, all counselling was provided by the primary investigator who had substantial clinical experience as a nurse in stroke care. This might have strengthened the quality of the counselling provided and have limited the amount of training required. It is uncertain how much training it would require if less experienced health professionals were to adapt the model.

Wearable activity trackers to monitor physical activity have become popular both in research and among common consumers, and evidence supports that they have a beneficial effect on physical activity in different populations [36–38]. We issued wearable activity trackers to participants in the intervention group to motivate the participants to physical activity. We met no noticeable resistance from the participants regarding using the device but identified several challenges. We had chosen a device with (1) long battery time so that the participants were not required to charge the device and (2) with a 4-week memory to limit the number of times the participants had to transfer data from the device to the smartphone application. The data transfer required an application to be downloaded and installed on a smartphone or tablet, the creation of a unique user account within the application, and the device to be connected to the application via Bluetooth. None of the participants was able to do this without support. When using technology as part of interventions provided to elderly and patients with neurological conditions, it is advisable to thoroughly consider possible limitations in terms of accessibility and technical skills of the participants and allocate resources to help and support.

### Limitations

By design, the sample size of this study was limited as the main purpose was to test the feasibility. We assumed, a priori, that a sample size of 40 participants would be enough to test all procedures of the study protocol. Overall, this number of participants proved to be sufficient in terms of testing the procedures and estimating rates of inclusion and attrition. It also helped us gain valuable experience on how to integrate this type counselling into a relatively compressed patient pathway.

The study progression was not evaluated using predefined progression criteria but through regular discussions with the steering committee. This pragmatic approach was decided due to the multimodal nature of the intervention, which could have made it difficult to foresee which elements were relevant to evaluate. Previous research on comparable study populations and interventions had, furthermore, reported substantial variations in rates of eligibility, acceptability, and attrition, making it difficult to define realistic criteria.

A general limitation of behavioural counselling interventions, as well as this study, is the limited possibility of blinding, making a large-scale RCT prone to potential bias with influence from social desirability.

Only three (7.5%) participants were current smokers, as opposed to 23% of Danish stroke patients being registered as current smokers [39], implying that smokers might be underrepresented.

To reduce the risk of information bias, data were collected according to a standardized study manual, and self-reported data were collected at baseline and follow-up using the same methods in both treatment groups.

### Implications for future research

In future research on health behaviour in patients with stroke, we need to address the prevalence of different health behaviours in more detail including the patients’ understanding of the causes of their stroke and awareness of risk, their readiness to change behaviour, and how patients who refuse to participate differ from participants, as alternative approaches might be needed to reach these patients.

## Conclusion

The study showed that despite several implementation challenges, it is within a narrow time frame of initial care possible to identify, recruit and randomize relevant patients early after admission, and retain most participants in the study until follow-up. Adjustments had to be made to the study protocol during the pilot period including the general omittance of the Astrand-Rhyming cycle test. The study was not designed to formally test the effect of the intervention on blood pressure, though the findings may serve as basis for designing large-scale randomized controlled trials.

### Supplementary Information

**Additional file 1: Additional Table 1.** Measures of effect (Secondary and tertiary outcomes).

**Additional file 2: Additional Table 2.** Activity tracker data presented per participant (3A) and per time interval (3B).

**Additional file 3: Additional Figure.** Boxplots of steps per day for each participant sorted by median. Each measurement is represented by an opaque grey square and darker colouring therefore signifies clustering of measurements. The dashed lines indicate the quartiles of all measurements.

## Data Availability

Data used during the current study will be available from the corresponding author upon reasonable request.
