# Supplementary material for: Lifestyle counselling as secondary prevention in patients with minor stroke or transient ischemic attack: a randomized controlled pilot study
Source: Pilot Feasibility Stud. 2024 Mar 22;10:50. doi: 10.1186/s40814-024-01478-4 (PMC10958836; doi:10.1186/s40814-024-01478-4)
Supplement: Supplementary file 1 — Additional file 1: Additional Table 1. Measures of effect (Secondary and tertiary outcomes). [file 40814_2024_1478_MOESM1_ESM.docx]

| **Additional table 1** Measures of effect (Secondary and tertiary outcomes) | | | | | |
| --- | --- | --- | --- | --- | --- |
|  | **Intention to treat** | |  | **Per protocol** | |
|  | **Intervention** | **Control** |  | **Intervention** | **Control** |
| **Arterial blood pressure** |  |  |  |  |  |
| **Systolic blood pressure** *(mmHg)* | n = 20 | n = 20 |  | n = 14 | n = 17 |
| Baseline ^A^ | 144.5 ± 14.71 | 140.5 ± 16.55 |  | 143.71 ± 12.98 | 141.58 ± 16.81 |
| 12-weeks follow-up ^A^ | 137.1 ± 14.84 | 133.5 ± 14.41 |  | 134.57 ± 12.34 | 131.88 ± 13.62 |
| Mean change ^A^ | -6.4 ± 9.3 | -8.3 ± 17.3 |  | -9.14 ± 10.0 | -9.70 ± 18.4 |
| Mean difference ^B^ |  | **1.85** [-7.13; 10.83] ^B^ |  |  | **0.56** [-10.16; 11.28] ^B^ |
|  |  |  |  |  |  |
| **Uncontrolled hypertension** *(SBP >140 mmHg)* | n = 20 | n = 20 |  | n = 14 | n = 17 |
| Baseline | 13 (65%) | 11 (55%) |  | 9 (73%) | 10 (53%) |
| 12-weeks follow-up | 7 (35%) | 6 (30%) |  | 3 (66%) | 5 (76%) |
| Difference ^C^ |  | **OR 1.0** [0.3; 3.9] ^C^ |  |  | **OR 1.5** [0.3; 6.8] ^C^ |
|  |  |  |  |  |  |
| **Diastolic blood pressure** | n = 20 | n = 20 |  | n = 14 | n = 17 |
| Baseline ^A^ | 86.1 ± 11.49 | 83.0 ± 11.32 |  | 87.93 ± 11.01 | 84.06 ± 11.61 |
| 12-weeks follow-up ^A^ | 81.35 ± 12.23 | 78.75 ± 8.74 |  | 81.14 ± 12.59 | 79.06 ± 8.98 |
| Mean change ^A^ | -4.75 ± 10.52 | -4.25 ± 10.84 |  | -6.79 ± 12.12 | -5.00 ± 11.65 |
| Mean difference ^B^ |  | **0. 5** [7.34; -6.34] ^B^ |  |  | **-1.78** [-10.6; 7.03] ^B^ |
|  |  |  |  |  |  |
| **Physical activity** |  |  |  |  |  |
| **MET-minutes per week** | n = 20 | n = 20 |  | n = 15 | n = 17 |
| Baseline ^A^ | 2809 ± 3732 | 1538 ± 1180 |  | 3606 ± 4009 | 1596 ± 1263 |
| 12-weeks follow-up ^A^ | 2048 ± 2242 | 1498 ± 801 |  | 2591 ± 2336 | 1594 ± 843 |
| Mean change ^A^ | -761 ± 3868 | -179 ± 1207 |  | -1015 ± 4476 | -211 ± 1312 |
| Difference ^B^ |  | -**582** [-2458; 1294] ^B^ |  |  | **-804** [-3344; 1736] ^B^ |
|  |  |  |  |  |  |
| **Time spent on physical activity** *(min/week)* | n = 20 | n = 20 |  | n = 15 | n = 17 |
| Baseline ^A^ | 765 ± 1071 | 344 ± 240 |  | 985 ± 1158 | 364 ± 255 |
| 12-weeks follow-up ^A^ | 537 ± 583 | 343 ± 193 |  | 681 ± 604 | 363 ± 202 |
| Mean change ^A^ | -228 ± 1130 | - 31 ± 234 |  | -304 ± 1306 | -36 ± 255 |
| Mean difference ^B^ |  | **-197** [-734; 340] ^B^ |  |  | **-268** [-999; 463] ^B^ |
|  |  |  |  |  |  |
| **Moderate/high level of physical activity** ^D^ | n = 20 | n = 20 |  | n = 15 | n = 17 |
| Baseline | 13 (65%) | 11 (55%) |  | 11 (73%) | 9 (53%) |
| 12-weeks follow-up | 12 (60%) | 15 (75%) |  | 10 (66%) | 13 (76%) |
| Difference ^C^ |  | **OR 0.5** [0.10; 2.32] ^C^ |  |  | **OR 0.62** [0.10; 3.78] ^C^ |
|  |  |  |  |  |  |
| **Body composition** |  |  |  |  |  |
| **Body weight** (kg) | n = 19 | n = 19 |  | n = 14 | n = 17 |
| Baseline ^A^ | 88.5 ± 15.46 | 82.6 ± 13.58 |  | 89.2 ± 13.82 | 83.4 ± 13.22 |
| 12-weeks follow-up ^A^ | 87.1 ± 13.18 | 80.6 ± 13.0 |  | 87.8 ± 10.20 | 81.2 ± 12.70 |
| Mean change ^A^ | -1.3 ± 5.1 | -1.9 ± 4.4 |  | -1.9 ± 6.1 | -2.2 ± 4.7 |
| Mean difference ^B^ |  | **-0.59** [-3.65; 2.47] ^B^ |  |  | **-0.37** [-4-47; 3.74] ^B^ |
|  |  |  |  |  |  |
| **Body Mass Index** | n = 19 | n = 19 |  | n = 14 | n = 17 |
| Baseline ^A^ | 28.6 ± 4.46 | 26.3 ± 3.48 |  | 28.5 ± 4.30 | 26.4 ± 3.59 |
| 12-weeks follow-up ^A^ | 28.2 ± 3.71 | 25.6 ± 3.31 |  | 27.9 ± 3.13 | 25.7 ± 3.43 |
| Mean change ^A^ | -0.4 ± 1.6 | -0.6 ± 1.5 |  | -0.6 ± 1.9 | -0.7 ± 1.6 |
| Mean difference ^B^ |  | **-0.2** [-1.18; 0.78] ^B^ |  |  | **-0.13** [-1.44; 1.18] ^B^ |
|  |  |  |  |  |  |
| **Waist-hip ratio** | n = 18 | n = 19 |  | n = 12 | n = 10 |
| Baseline ^A^ | 1.0 ± 0.09 | 0.98 ± 0.11 |  | 1.01 ± 0.1 | 0.99 ± 0.1 |
| 12-weeks follow-up ^A^ | 0.99 ± 0.07 | 0.97 ± 0.11 |  | 1.00 ± 0.07 | 0.97 ± 0.09 |
| Mean change ^A^ | -0.01 ± 0.03 | -0.01 ± 0.05 |  | -0.01 ± 0.04 | -0.03 ± 0.07 |
| Mean difference ^B^ |  | **0.0** [-0.02; 0.03] ^B^ |  |  | **0.01** [-0.04; 0.06] ^B^ |
|  |  |  |  |  |  |
| **Fatigue** | n = 19 | n = 20 |  | n = 15 | n = 17 |
| Baseline ^A^ | 18.9 ± 6.15 | 16.2 ± 4.58 |  | 18.4 ± 6.67 | 14.9 ± 1.96 |
| 12-weeks follow-up ^A^ | 20.3 ± 8.27 | 19.9 ± 7.46 |  | 20.2 ± 9.23 | 19.2 ± 7.36 |
| Mean change ^A^ | 1.42 ± 4.21 | 3.70 ± 7.45 |  | 1.80 ± 4.69 | 4.35 ± 7.93 |
| Mean difference ^B^ |  | **2.27** [-1.65; 6.21] ^B^ |  |  | **2.55** [-2.12; 7.22] ^B^ |
|  |  |  |  |  |  |
| **Long-term follow-up** *(1 year)* | n = 20 | n = 20 |  | n = 17 | n = 19 |
| Recurrent stroke | 1 (5%) | 1 (5%) |  | 1 (5.9%) | 1 (5.3%) |
| Other vascular events | 0 | 0 |  | 0 | 0 |
| Fatalities | 0 | 0 |  | 0 | 0 |
| **MET** Metabolic Equivalents, ^A^ mean ± standard deviation, ^B^ mean difference [95% confidence interval], ^C^ odds ratio [95% confidence interval]  ^D^ Combination of walking, moderate or vigorous intensity activities achieving a total physical activity of at least 600 MET minutes a week  Continuous outcome measures were compared between groups using change scores from baseline to follow-up and reported as mean difference with a symmetrical 95% confidence interval based on t-distributions. Confidence intervals of all proportions were calculated using the Exact method (Clopper-Pearson). Logistic regression was used to compare the odds of a more favourable outcome of categorical outcome measures at follow-up than at baseline. Differences between allocation groups were tested using an intention-to-treat model, with last value carried over when follow-up data were missing [30]. Per protocol analysis included all participants who received initial counselling and attended re-evaluation after 12 weeks. P-values are not reported because the study was not powered for formal hypothesis testing. | | | | | |
