# Supplementary material for: Lifestyle counselling as secondary prevention in patients with minor stroke or transient ischemic attack: a randomized controlled pilot study
Source: Pilot Feasibility Stud. 2024 Mar 22;10:50. doi: 10.1186/s40814-024-01478-4 (PMC10958836; doi:10.1186/s40814-024-01478-4)
Supplement: Supplementary file 2 — Additional file 2: Additional Table 2. Activity tracker data presented per participant (3A) and per time interval (3B). [file 40814_2024_1478_MOESM2_ESM.docx]

| **Additional table 2** Activity tracker data presented per participant (3A) and per time interval (3B) | | | |
| --- | --- | --- | --- |
|  | **Steps per day^A^** | **Aerobic walking time^A^** | **Adherence^B^** |
| **3A Participant** (sorted from lowest to highest median) | | | |
| A | 3606 ± 1861 | 4.72 ± 12.11 | 62.4% [51.2 - 72.6] |
| B | 4358 ± 2077 | 3.32 ± 10.99 | 73.8% [63.1 - 82.8] |
| C | 4096 ± 1652 | 0.16 ± 1.52 | 100% [95.8 - 100] |
| D | 4833 ± 2218 | 34.77 ± 21.85 | 100% [96.0 - 100] |
| E | 7066 ± 4225 | 23.67 ± 33.49 | 98.8% [93.6 - 100] |
| F | 8884 ± 3079 | 8.45 ± 12.02 | 62.9% [52.9 - 72.1] |
| G | 8585 ± 3118 | 30.90 ± 27.77 | 96.5% [90.1 - 99.3] |
| H | 9572 ± 2455 | 1.76 ± 4.34 | 95.6% [89.0 - 98.8] |
| I | 10068 ± 3124 | 13.02 ± 24.14 | 100% [95.7 - 100] |
| J | 11897 ± 8597 | 61.40 ± 60.96 | 100% [95.7 - 100] |
| K | 11168 ± 5322 | 10.37 ± 16.53 | 95.3% [88.4 - 98.7] |
| L | 13024 ± 1781 | 53.06 ± 18.38 | 97.8% [92.3 - 99.7] |
| **3B Time** |  |  |  |
| T1: weeks 1-3 | 7282 ± 3998 | 16.80 ± 24.23 | 91.7% [87.5 - 94.8] |
| T2: weeks 4-6 | 8809 ± 4641 | 24.70 ± 34.10 | 86.1% [81.2 - 90.1] |
| T3: weeks 7-9 | 8851 ± 4970 | 24.54 ± 35.56 | 86.9% [82.1 - 90.8] |
| T4: weeks 10-12 | 7941 ± 5089 | 20.60 ± 35.22 | 98.4% [96.0 - 99.6] |
| ^A^ mean ± standard deviation  ^B^ Percentage of days patients wore the VivoFit; 95% confidence intervals were calculated using the Exact-method | | | |
