# Supplementary material for: Lifestyle counselling as secondary prevention in patients with minor stroke or transient ischemic attack: a randomized controlled pilot study
Source: Pilot Feasibility Stud. 2024 Mar 22;10:50. doi: 10.1186/s40814-024-01478-4 (PMC10958836; doi:10.1186/s40814-024-01478-4)
Supplement: Supplementary file 3 — Additional file 3: Additional Figure. Boxplots of steps per day for each participant sorted by median. Each measurement is represented by an opaque grey square and darker colouring therefore signifies clustering of measurements. The dashed lines indicate the quartiles of all measurements. [file 40814_2024_1478_MOESM3_ESM.docx]

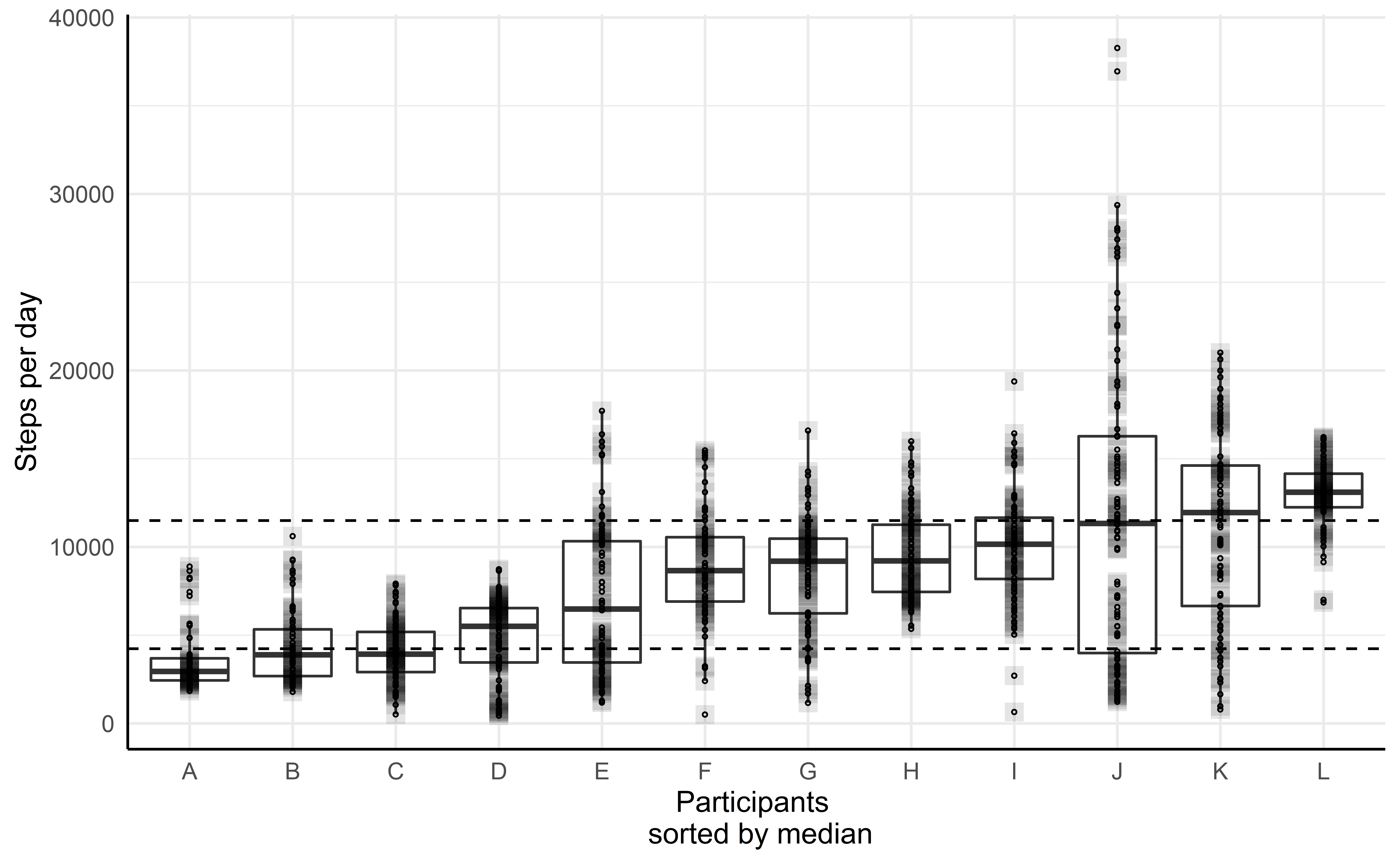


**Additional figure** Boxplots of steps per day for each participant sorted by median. Each measurement is represented by an opaque grey square and darker colouring therefore signifies clustering of measurements. The dashed lines indicate the quartiles of all measurements.
